# 

**Published:** 1882-10

**Authors:** 


### CONTENTS:

PAGE.

Original Communications:

Histology of the Nerves; a Contribution of the Latest Researches. By Frank Dudley Beane, A. M., M. D., . . . . 97

Arsenical Poisoning—The "Kloss Case"—Unusual Post-mortem Appearances. By A. R. Davidson, M. D., . . . . . 117

Clinical Reports:

Two Cases of Simple Fracture of the Skull Unrecognized for Days—Late Appearance of Symptoms—Trephining—Results. Reported by J. H. Pryor, M. D., 119

Conservative Surgery. By P. W. Van Peyma, M. D., . . . . . . 121

Selections:

Bright's Disease. By Alonzo Clark, M. D., 123

Society Reports:

Buffalo Medical and Surgical Association, . 130

Editorial:

The Buffalo State Asylum for the Insane—Annual Report, . . . . . . 136

The American Pharmaceutical Association, 139

Buffalo Medical College—Session of 1882-83, 141

Editorial Notes, . . . . . . . 142

Reviews: . . . . . . . 143

